# Autophagy modulating therapeutics inhibit ovarian cancer colony generation by polyploid giant cancer cells (PGCCs)

**DOI:** 10.1186/s12885-022-09503-6

**Published:** 2022-04-14

**Authors:** Robert R. Bowers, Maya F. Andrade, Christian M. Jones, Shai White-Gilbertson, Christina Voelkel-Johnson, Joe R. Delaney

**Affiliations:** 1 Department of Biochemistry and Molecular Biology, Medical University of South Carolina, Charleston, SC USA; 2 Department of Microbiology and Immunology, Medical University of South Carolina, Charleston, SC USA

**Keywords:** Aneuploidy, Autophagy, Cancer recurrence, Chemoresistance, Neosis, Ovarian cancer, Polyploid giant cancer cells (PGCCs), Senescence, Whole genome doubling

## Abstract

**Background:**

Genomic instability and chemoresistance can arise in cancer due to a unique form of plasticity: that of polyploid giant cancer cells (PGCCs). These cells form under the stress of chemotherapy and have higher than diploid chromosome content. PGCCs are able to then repopulate tumors through an asymmetric daughter cell budding process. PGCCs have been observed in ovarian cancer histology, including the deadly and common form high-grade serous ovarian carcinoma (HGSC). We previously discovered that drugs which disrupt the cellular recycling process of autophagy are uniquely efficacious in pre-clinical HGSC models. While autophagy induction has been associated with PGCCs, it has never been previously investigated if autophagy modulation interacts with the PGCC life cycle and this form of tumor cell plasticity.

**Methods:**

CAOV3 and OVCAR3 ovarian cancer cell lines were treated with carboplatin or docetaxel to induce PGCC formation. Microscopy was used to characterize and quantify PGCCs formed by chemotherapy. Two clinically available drugs that inhibit autophagy, hydroxychloroquine and nelfinavir, and a clinically available activator of autophagy, rapamycin, were employed to test the effect of these autophagy modulators on PGCC induction and subsequent colony formation from PGCCs. Crystal violet-stained colony formation assays were used to quantify the tumor-repopulating stage of the PGCC life cycle.

**Results:**

Autophagy inhibitors did not prevent PGCC formation in OVCAR3 or CAOV3 cells. Rapamycin did not induce PGCC formation on its own nor did it exacerbate PGCC formation by chemotherapy. However, hydroxychloroquine prevented efficient colony formation in CAOV3 PGCCs induced by carboplatin (27% inhibition) or docetaxel (41% inhibition), as well as in OVCAR3 cells (95% and 77%, respectively). Nelfinavir similarly prevented colony formation in CAOV3 PGCCs induced by carboplatin (64% inhibition) or docetaxel (94% inhibition) as well as in OVCAR3 cells (89% and 80%, respectively). Rapamycin surprisingly also prevented PGCC colony outgrowth (52–84% inhibition).

**Conclusions:**

While the autophagy previously observed to correlate with PGCC formation is unlikely necessary for PGCCs to form, autophagy modulating drugs severely impair the ability of HGSC PGCCs to form colonies. Clinical trials which utilize hydroxychloroquine, nelfinavir, and/or rapamycin after chemotherapy may be of future interest.

**Supplementary Information:**

The online version contains supplementary material available at 10.1186/s12885-022-09503-6.

## Background

Ovarian cancer is the fifth most frequent cause of cancer death in women. It is estimated that 21,410 new cases and 13,770 deaths occurred in the United States in 2021 [1]. Ovarian cancer is a heterogenous disease that is classified into serous, endometroid, mucinous, and clear cell subtypes based on distinct histology and genetics. Ovarian high-grade serous carcinoma (HGSC) is the most common and deadly histotype and is responsible for approximately 70% of ovarian cancer cases and deaths [2, 3]. This most lethal female reproductive cancer is nicknamed the “silent killer” as patients are frequently diagnosed at advanced stages with metastatic disease [4]. The standard-of-care for HGSC patients includes cytoreductive surgery and chemotherapy, usually carboplatin and paclitaxel, but many patients experience platinum-resistant relapse and 5-year survival rates are less than 50% [1]. While cancer research has seen profound progress in many areas over the past five decades, only marginal increases in overall- and disease-free survival in ovarian cancer patients have occurred and improved therapies are critically needed.

Ploidy is more flexible in cancer cells than non-transformed cells. HGSC tumors are triploid or higher in whole-genome average ploidy in 53–56% of cases [5, 6]. Single-cell sequencing has revealed untreated on-average diploid HGSC tumors exhibit 2–4% of epithelial cells in a triploid or higher ploidy [7]. Ploidy gains can be induced in cancer cells, often by chemotherapy or other forms of stress. Following a lethal dose of chemotherapy, most cells in a population undergo cell death, but some cells are able to enter a quiescent, therapy-induced state and survive. Remarkably, many of these cells have been observed in histological sections to be polyploid, including in ovarian cancer [8]. Such polyploid cells may contain either a single much-enlarged nucleus or an amalgamation of diploid or larger sized nuclei. Polyploid giant cancer cells (PGCCs) are defined as cancer cells with tetraploid or higher ploidies (4 N) that express markers of or have properties of stem cells [9–12].

PGCCs exhibit unique life cycle characteristics which implicate their important roles in chemoresistance and tumor evolution. Polyploidy initially forms by a variety of mechanisms which can include cell–cell fusion or endoreplication (duplication of the genome without mitosis). Chemotherapies such as DNA-damaging platinum agents and microtubule-stabilizing taxanes induce formation of PGCCs. These PGCCs are temporarily arrested in the cell cycle and express senescence markers such as p21 [13]. After a period of days to weeks, PGCCs re-enter the cell cycle and repopulate the tumor with drug-resistant progeny [14, 15]. This can occur partially through symmetric division of polyploid cells, but more substantially occurs via asymmetric budding of lower-ploidy daughter cells from the originating PGCC, which then re-enter the cell cycle. The latter daughter-budding process is termed “neosis,” which we adopt here [11]. As the PGCC progeny resemble the original parental cells, the entire process of PGCC formation and subsequent progeny generation is referred to as the PGCC life cycle [9, 10]. PGCC progeny are resistant to the therapies that originally induced their formation, recapitulating the development of drug-resistant cancers [12, 13, 16]. Progeny of ovarian cancer PGCCs have highly variable karyotypes, providing a source of genetic diversity which may enable the evolution of chemoresistance [17].

Aneuploidy is unusually high in HGSC. HGSC has ~ 16,000 genes altered in dosage by copy number alterations (CNAs) in the median tumor due to a high degree of aneuploidy and focal (sub chromosome arm-level) copy number alterations [18]. Specifically, the tumor suppressor p53 is mutated in essentially all (96%) HGSCs [19, 20], enabling aneuploid cells to survive. Using genetic pathway analyses of HGSC CNAs, we discovered the autophagy cellular recycling pathway is the most downregulated pathway by CNA losses with 98% of tumors having multiple heterozygous deletions of autophagy genes. Yet, autophagy remains critical for these cancer cells. Autophagy is a stress response mechanism required for drug resistance in ovarian cancer [21–23]. Autophagy is upregulated during the formation of PGCCs [24, 25].

We previously discovered that autophagy is a targetable vulnerability, as drugs disrupting autophagy killed both chemo-sensitive and chemo-resistant ovarian cancer cells *in vitro* and *in vivo* [18, 26]. HGSC growth was inhibited by autophagy inhibitors, chloroquine or nelfinavir, as well as autophagy inducers, such as the mTORC1 inhibitor rapamycin. However, neither we nor the PGCC field has examined whether autophagy drugs impinge on the life cycle of PGCCs. We hypothesized that autophagy-modulating-therapeutics may interfere with the chemotherapy-induced PGCC life cycle in ovarian cancer cells. Here, the impact of these autophagy modulators on chemotherapy-induced PGCC formation and neosis by PGCCs was investigated.

## Methods

### Cell culture

CAOV3 and OVCAR3 human ovarian cancer cell lines were from ATCC and were cultured in RPMI-1640 supplemented with L-glutamine, 10% fetal bovine serum, sodium pyruvate, and penicillin–streptomycin. Cells were incubated at 37 °C with 5% CO_2_.

### Chemotherapy-induced polyploid giant cancer cell induction and neosis

Carboplatin- and docetaxel-induced PGCC formation and subsequent daughter cell formation were studied over the span of 14 days. CAOV3 cells were seeded at 100,000 cells/mL and OVCAR3 cells were seeded at 250,000 cells/mL, 24 h later cells were treated with 10 µM carboplatin or 5 nM docetaxel for 3 days, followed by 3 days of recovery. For experiments testing effects of autophagy-targeting therapeutics on PGCC development, cells were treated with 33 µM hydroxychloroquine, 10 µM nelfinavir, or 10 nM rapamycin alone or concurrently with carboplatin or docetaxel, and after 3 days of drug treatments and 3 days of recovery, cells were fixed, stained, imaged, and nuclear content was quantified as described below. For studies of daughter cell formation by PGCCs, on day 7 PGCCs were isolated based on size-exclusion with pluriSelect™ cell strainers of 30 µm for CAOV3 cells and 10 µm for OVCAR3 cells. Then cells were re-plated, allowed to rest for 24 h, and treated with 33 µM hydroxychloroquine, 10 µM nelfinavir, or 10 nM rapamycin for a total of six days with a media change containing fresh drugs in the middle. Finally, colonies which arose from PGCCs were fixed, imaged, and quantified through crystal violet staining as described below.

### Nuclear Quantification

DNA staining with Hoechst 33342 was used to quantify changes in CAOV3 and OVCAR3 cell nuclear content. Specifically, cells were fixed with ice-cold methanol at -20 °C for 7 min, permeabilized with 0.1% Triton X-100 for 2 min, blocked with 5% bovine serum albumin (BSA) / 5% goat serum in phosphate buffer saline (PBS) at room temperature for 45 min, and incubated with the primary antibody mouse anti-E-Cadherin (BD, #610182) overnight. Secondary anti-rabbit Alexa Fluor 594 (Fisher Scientific) was used at 1:1,000 and Hoechst 33342 (Fisher Scientific, #A11029) was used at 1:10,000 and were diluted into 5% BSA / 5% goat serum and incubated for 90 min. The immunofluorescent cells were then imaged using the Lionheart FX automated microscope (BioTek) and NIH ImageJ (Fiji) software was utilized in addition with a custom macro to measure nuclear area and intensity using Hoechst 33342 staining. Fifty representative cells were counted in each of two independent experiments, and the data were normalized and aggregated. The median nuclear area X intensity of the control CAOV3 and OVCAR3 cells was designated as “normal ploidy”, and to exclude cells undergoing normal mitotic processes (normal—2X normal ploidy), a threshold DNA content ≥ 4.5X normal ploidy was used to classify cells as PGCCs. Using the total number of cells classified as “normal” or PGCCs, contingency tables were generated, and Fisher’s exact tests were conducted to test for significant differences between treatment groups.

### Western blotting

Western blotting was performed as described previously [18] to confirm that autophagy-targeting therapeutic treatment affected the expression of the autophagy markers GRP78 and LC3B-II. As above, CAOV3 cells were treated with 33 µM hydroxychloroquine, 10 µM nelfinavir, or 10 nM rapamycin alone or concurrently with carboplatin or docetaxel for 72 h, then cells were lysed in ice-cold RIPA buffer supplemented with a protease inhibitor cocktail (Sigma-Aldrich). After centrifugation at 10,000 g for 10 min at 4 °C, protein concentration in the supernatants was quantified by bicinchoninic acid assay (BCA; Pierce #23235). For each sample, 30 µg was resolved on 4 – 20% gradient polyacrylamide gels (Biorad #4561093), transferred to nitrocellulose membranes, blocked using 5% milk in PBS, and incubated overnight with β-actin (Thermo Fisher #MA515739), GRP78 (Cell Signaling #3177), or LC3B (Novus Biologicals #NB100-2220) primary antibodies at 1:1000 dilutions. Horseradish peroxidase conjugated goat anti-mouse (Sigma #12–3349) and goat anti-rabbit (VWR #100244–772) secondary antibodies were incubated in TBST at 1:5000 dilutions for 45 min. Enhanced chemiluminescence (ECL, Biorad #1705060), a Chemidoc Imaging System (Biorad), and NIH ImageJ software were used to visualize the results.

### Quantification of colony formation with crystal violet

PGCCs were isolated after three days of chemotherapy treatment followed by three days of rest. Specifically, CAOV3 and OVCAR3 cells were trypsinized and filtered through 30 µm size-exclusion cell strainers for CAOV3 cells (pluriSelect, #43-50030-03) and with cell strainers of 10 µm size-exclusion for OVCAR3 cells (pluriSelect, #43-50010-03). After rinsing the strainers with 10 mL of media, the cell strainers were inverted and PGCCs were gathered and re-plated. After one week of colony outgrowth, CAOV3 and OVCAR3 cells were fixed in methanol, stained with 0.2% crystal violet in PBS at room temperature for 20 min, washed twice with PBS, and representative brightfield images were acquired with a Lionheart FX microscope (BioTek). After imaging, crystal violet was resuspended in 10% glacial acetic acid and absorbance at 600 nm was read in an Epoch 2 spectrophotometer (BioTek).

### Statistical analysis

For the data in PGCC formation assays, statistical significance was calculated using Fisher’s exact tests. For the data in colony quantitation assays, a two-tailed, Student’s *t*-test was used to calculate statistical significance. *P* < 0.05 was considered statistically significant.

## Results

### PGCC life cycle overview and experimental setup

**Fig. 1:**
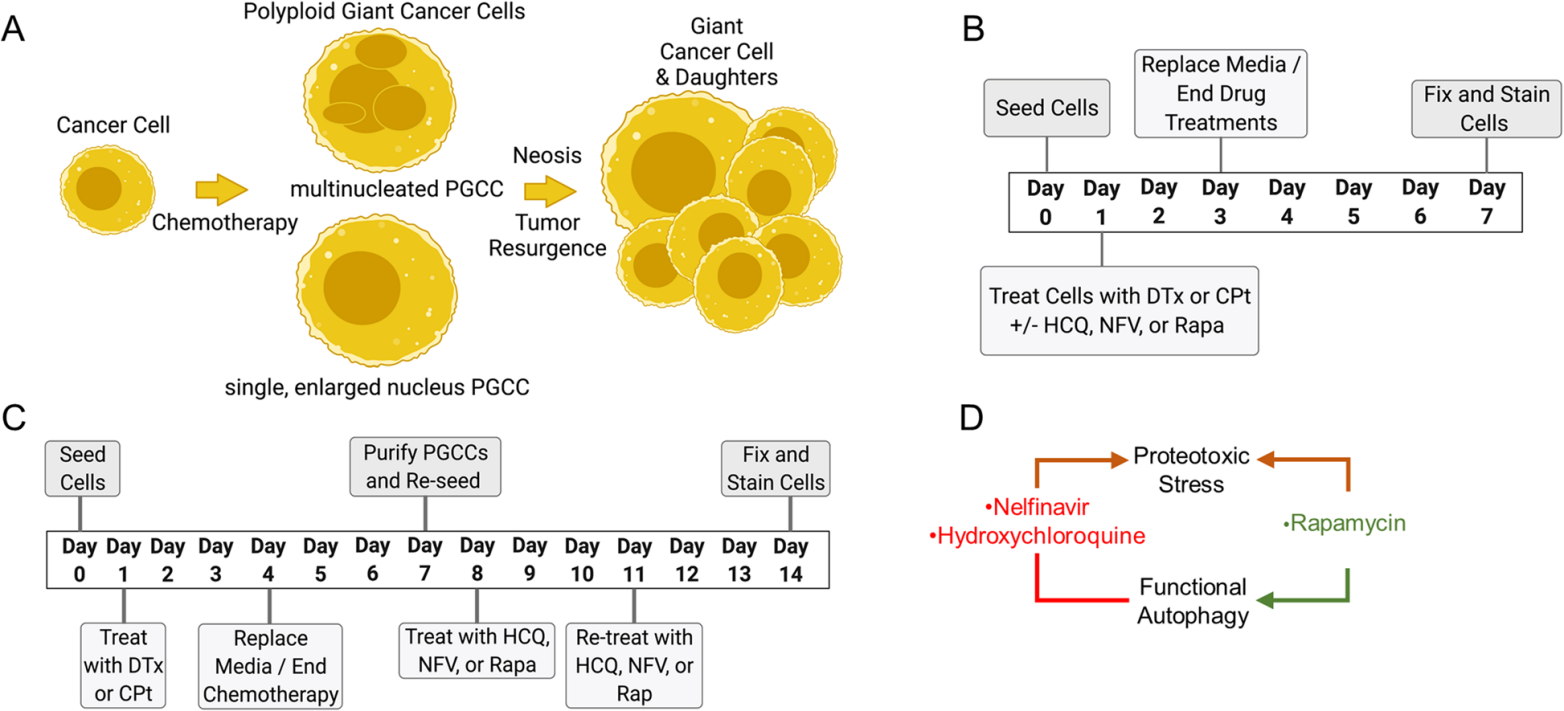
Polyploid Giant Cancer Cell (PGCC) life cycle and experimental setup for CAOV3 and OVCAR3 PGCC induction and subsequent progeny generation.** A** PGCC life cycle depicting a diploid cancer cell undergoing chemotherapy-induced polyploidization followed by neosis – the generation of diploid progeny through depolyploidization. **B** Timeline for assessing the effect of the autophagy modulators hydroxychloroquine (HCQ), nelfinavir mesylate (NFV), and rapamycin (Rapa) on carboplatin- (CPt) or docetaxel- (DTx) induced PGCC formation and subsequent progeny generation. CAOV3 or OVCAR3 cells were seeded on day 0, treated with chemotherapy drugs CPt or DTx from day 1 through day 4, allowed to recover for three days. To assess the effect of the autophagy modulators on PGCC formation, cells were treated with HCQ, NFV, or Rapa concurrently with CPt or DTx, then fixed, stained, and imaged on day 7 as described in Methods. **C** To assess the effect of the autophagy modulators on PGCC progeny development, cells were treated with CPt or DTx from day 1 through day 4 and allowed to recover from day 4 – day 7 as above, but then PGCCs were separated based on cell size and re-plated, allowed to adhere overnight, then treated with HCQ, NFV, or Rapa on day 8 through day 14 – a time during which PGCCs form progeny. Finally, PGCC progeny were fixed, stained with crystal violet, imaged, and quantified as described in Methods. **D** Clinically available autophagy drugs used in this study are briefly diagrammed for their mechanisms in terms of proteotoxic stress and modulation of functional autophagy

The PGCC life cycle is illustrated in Fig. 1A: a cancer cell undergoes polyploidization due to genotoxic or microtubule stress caused by chemotherapy to form a PGCC, which can have multiple nuclei or a single, enlarged nucleus. Adopting the polyploid state allows the PGCC to survive the stress, and following resolution of the stress, PGCCs eventually produce progeny through neosis. Two sets of experiments were conducted to assay the interaction of autophagy drugs with this PGCC life cycle: the first set focused on PGCC generation (Fig. 1B), whereas the second set investigated progeny generation by PGCCs (Fig. 1C). Note that PGCCs cells are assessed (day 7) after many diploid cells have died from carboplatin (CPt) or docetaxel (DTx) chemotherapy (peaking on days 3–5). As this study seeks to understand the interaction of autophagy with the PGCC life cycle, three autophagy-modulating drugs were used: hydroxychloroquine, nelfinavir mesylate, and rapamycin (Fig. 1D). Nelfinavir mesylate (NFV) induces endoplasmic reticulum stress [27], but also inhibits autophagy [18]. Hydroxychloroquine (HCQ), a clinically used derivative of chloroquine, prevents autophagosome-lysosome fusion, creating proteotoxicity while also inhibiting autophagic flux [28]. Rapamycin (Rapa), an mTORC1 inhibitor, induces autophagy by de-repressing ULK1 [29], but also slows cellular proliferation and creates ribosomal imbalances [30]. All three were derived from our previous drug studies showing each independently contribute to cellular toxicity in HGSC cells *in vitro* and *in vivo* [18, 26].

### Induced formation of PGCCs by standard HGSC chemotherapies

**Fig. 2:**
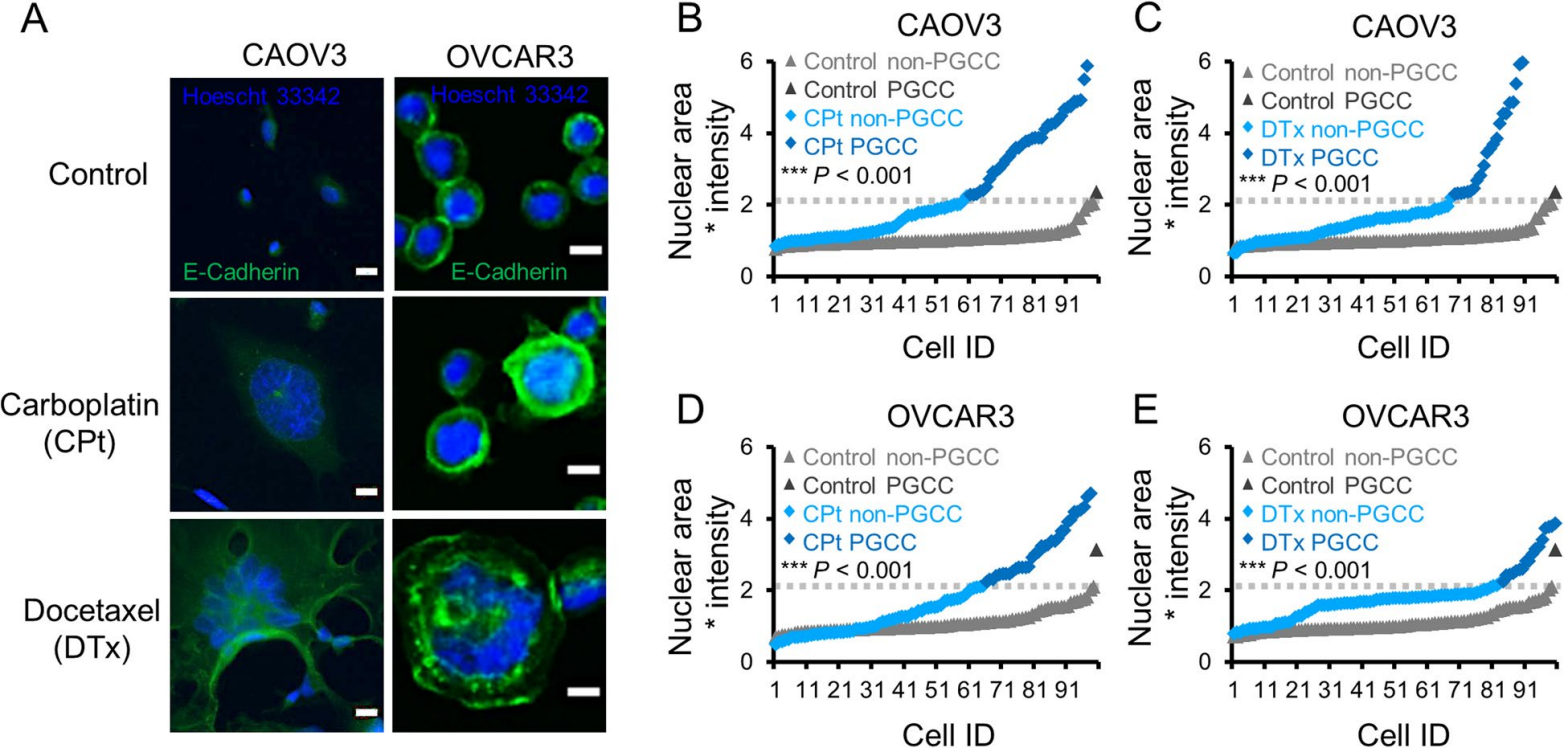
Carboplatin (CPt) and docetaxel (DTx) induce the formation of CAOV3 and OVCAR3 PGCCs. **A** Representative photomicrographs of CAOV3 and OVCAR3 PGCCs formed by 10 µM CPt or 5 nM DTx are shown. E-Cadherin stain (green) was used to allow cytoplasmic determination, while Hoescht 33342 (blue) was used for nuclei quantitation. Note E-Cadherin well delineates cell–cell junctions in bound OVCAR3 cells. Scale bars are 10 µm. **B** CAOV3 cells were treated with 10 µM CPt or vehicle control (72 h treatment) and assessed for PGCC formation (72 h after CPt removal, by Hoescht 33342 quantitation). Threshold for PGCC identification is indicated by dashed gray line. **C** CAOV3 cells were similarly assessed for PGCC formation induced by 5 nM DTx. **D**, **E** OVCAR3 cells were similarly assessed for PGCC formation rates. *P*-values indicated Fisher’s exact test result, and 100 cells quantified from two independent experiments are shown

OVCAR3 and CAOV3 cell lines were chosen as models because they have two hallmarks of HGSC: both have p53 mutations and extensive CNAs [31]. To generate PGCCs, CAOV3 or OVCAR3 cells were seeded on day 0, treated with 10 µM CPt or 5 nM Dtx for 72 h (days 1–4) and then allowed to recover for 72 h before ending the experiment on day 7 (see Fig. 1B). DNA content in 100 untreated, CPt-, and DTx-treated cells from two independent experiments was quantified in both CAOV3 and OVCAR3 cells after 3 days of chemotherapy and 3 days of rest. Example images of PGCCs are provided in Fig. 2A. The median DNA content of the control cells was defined as “normal ploidy” and, to exclude cells undergoing normal mitotic processes (normal to 2X normal), cells having ≥ 4.5X normal DNA content were classified as PGCCs as described in Methods. Contingency tables were generated using the total number of cells classified as “normal” or PGCCs, and Fisher’s exact tests were conducted to test for significant differences between treatment groups. In untreated CAOV3 cells, 1% of cells were classified as PGCCs whereas CAOV3 cells treated with CPt contained 40% PGCCs (Fig. 2B) and DTx treated cells contained 32% PGCCs (Fig. 2C). For untreated OVCAR3 cells, 1% of cells were classified as PGCCs whereas OVCAR3 cells treated with CPt or DTx had 16% and 35% PGCCs, respectively (Fig. 2D and E). These data demonstrate that both CPt and DTxl induce the formation of PGCCs in CAOV3 and OVCAR3 ovarian cancer cell lines. These polyploid cells had DNA contents of 4.5X – 16X normal or more.

### Autophagy inhibitors do not prevent PGCC formation

**Fig. 3:**
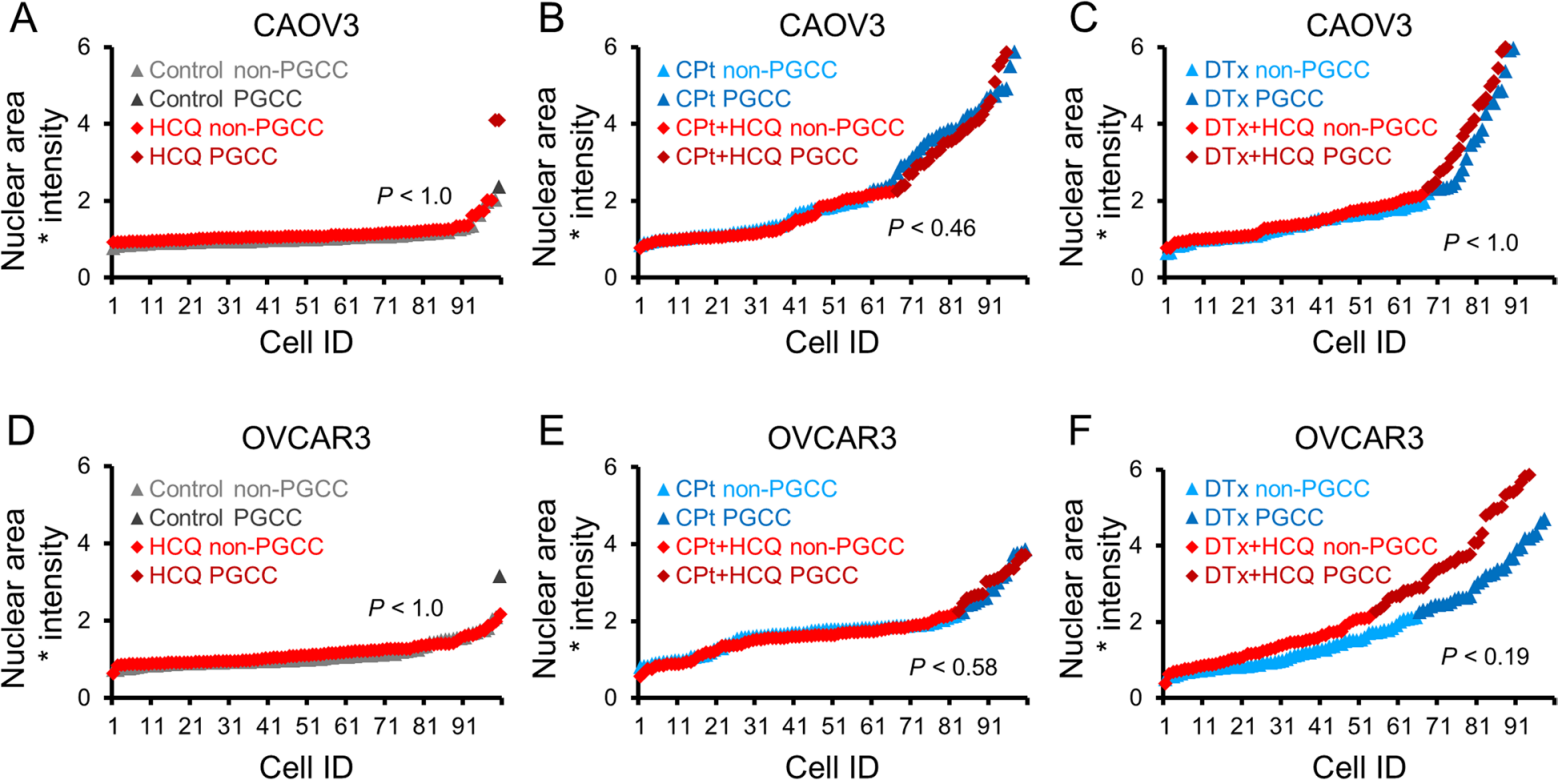
Hydroxychloroquine (HCQ) does not prevent CPt-or DTx–induced PGCC formation in CAOV3 and OVCAR3 cells.** A** CAOV3 cells were treated with 33 µM HCQ or vehicle control for 72 h and allowed to recover in fresh drug-free media for 72 h prior to PGCC quantitation by Hoescht 33342 staining. **B** CAOV3 cells were co-treated with CPt and HCQ or vehicle control for 72 h and allowed to recover in fresh drug-free media for 72 h prior to PGCC quantitation. **C** CAOV3 cells were co-treated with DTx and HCQ or vehicle control for 72 h and allowed to recover in fresh drug-free media for 72 h prior to PGCC quantitation. **D**-**F** Identical experiments as in (**A-C**) were performed using OVCAR3 cells. *P*-values indicated Fisher’s exact test result, and 100 cells quantified from two independent experiments are shown

Given that autophagy is known to be upregulated during PGCC formation [24, 25], we next investigated if clinically available inhibitors of autophagy prevented chemotherapy-induced PGCC formation. The inhibitors HCQ or NFV were used. CAOV3 or OVCAR3 cells were treated with HCQ alone or concurrently with CPt or DTx for 3 days (day 1 – day 4) and allowed to recover for 3 days. Cells were fixed on day 7 and nuclear area and intensity were quantified as previously described. CAOV3 cells treated with HCQ alone had 2% PGCCs, a non-significant difference than the 1% PGCCs observed in control cells (Fig. 3A). Although concurrent treatment of CAOV3 cells with HCQ and CPt decreased PGCCs to 34% versus 40% in CPt alone, this trend was not statistically significant (Fig. 3B). There was no difference in CAOV3 cells co-treated with HCQ and DTx compared to DTx alone, 33% versus 32% PGCCs respectively (Fig. 3C). OVCAR3 cells treated with HCQ alone and control OVCAR3 cells both had 1% PGCCs (Fig. 3D). Concurrent treatment of OVCAR3 cells with HCQ and CPt yielded slightly, but not significantly more PGCCs than CPt alone, 20% versus 16%, respectively (Fig. 3E). Similarly, OVCAR3 cells co-treated with HCQ and DTx had 45% PGCCs compared to 35% PGCCs for DTx alone, but this increase was not significant (*P* = 0.19; Fig. 3F). Overall, these data show that co-treatment with HCQ and chemotherapy led to similar levels of PGCC induction as chemotherapy alone in CAOV3 and OVCAR3 cells.

**Fig. 4:**
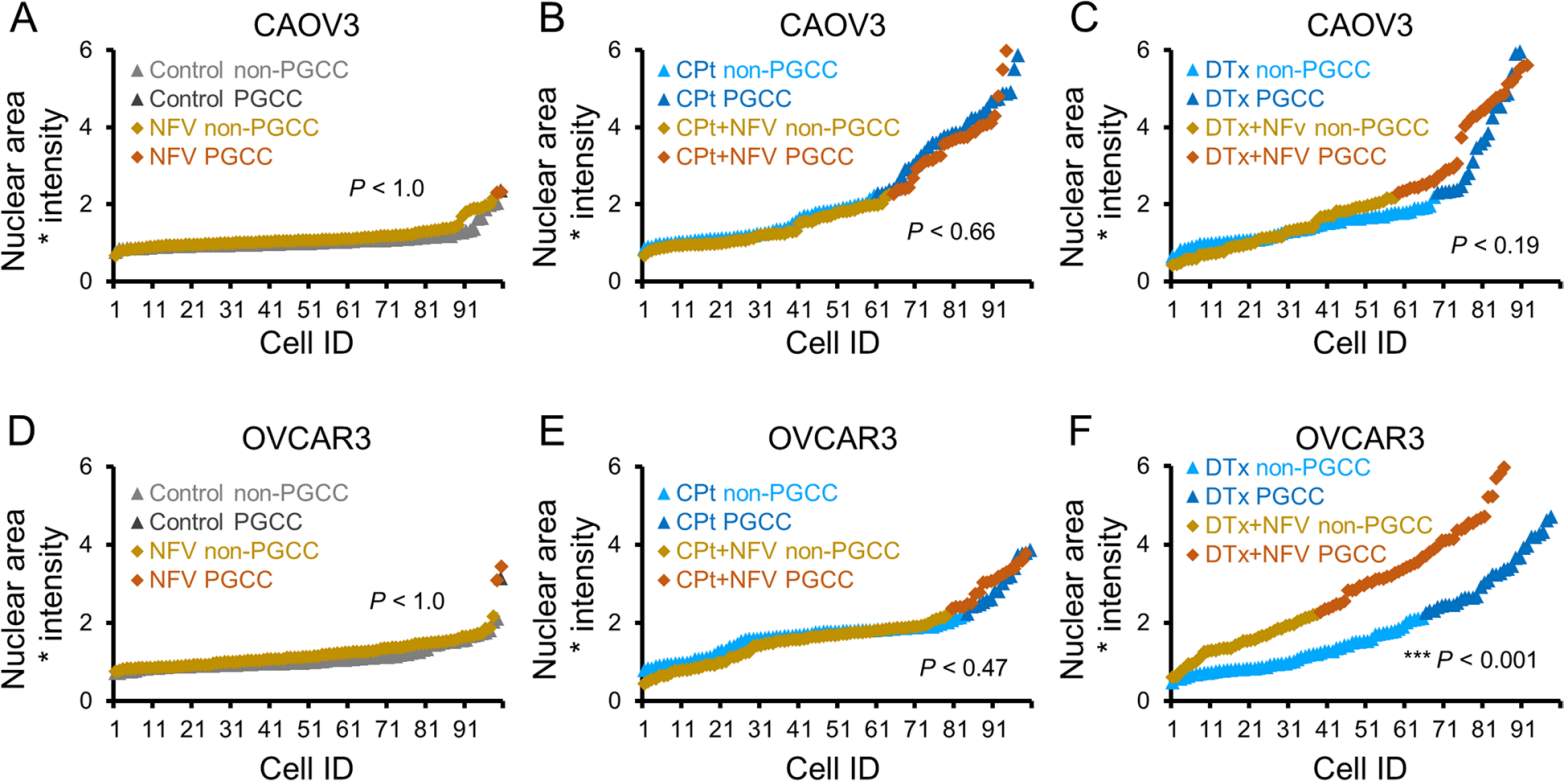
Nelfinavir mesylate (NFV) does not prevent CPt- or DTx–induced PGCC formation in CAOV3 or OVCAR3 cells.** A** CAOV3 cells were treated with 10 µM NFV or vehicle control for 72 h and allowed to recover in fresh drug-free media for 72 h prior to PGCC quantitation by Hoescht 33342 staining. **B** CAOV3 cells were co-treated with CPt and NFV or vehicle control for 72 h and allowed to recover in fresh drug-free media for 72 h prior to PGCC quantitation. **C** CAOV3 cells were co-treated with DTx and NFV or vehicle control for 72 h and allowed to recover in fresh drug-free media for 72 h prior to PGCC quantitation. **D-F** Identical experiments as in (**A-C**) were performed using OVCAR3 cells. *P*-values indicated Fisher’s exact test result, and 100 cells quantified from two independent experiments are shown

Next, NFV was tested in the context of chemotherapy-induced PGCC formation. NFV alone had no effect on PGCC incidence in CAOV3 cells, 2% PGCCs were observed following NFV versus 1% PGCCs in control cells (Fig. 4A). In CAOV3 cells co-treated with CPt and NFV there were 36% PGCCs compared to 40% PGCCs in CPt alone, not a significant difference (Fig. 4B). Although there was a trend for co-treatment of NFV and DTx to increase PGCC formation compared to DTx alone to 42% from 32% in CAOV3 cells, this trend was not statistically significant (Fisher’s exact test statistic 0.19; Fig. 4C). As in CAOV3 cells, NFV treatment alone had no effect on background PGCC incidence in OVCAR3 cells, 2% PGCCs in NFV-treated versus 1% PGCCs in the control cells (Fig. 4D). In CPt treated OVCAR3 cells, NFV co-treatment had 21% PGCCs versus 16% PGCCs in CPt alone, an insignificant increase (Fig. 4E). In OVCAR3 cells co-treated with DTx and NFV compared to DTx alone, however, a marked increase to 60% from 35% PGCCs was observed (*p* = 0.0006; Fig. 4F). Thus, in both CAOV3 and OVCAR3 cells, NFV caused no changes in basal PGCC levels or in PGCC levels induced by CPt. In the context of DTx-induced PGCC formation, there was a trend for NFV to increase PGCC formation by CAOV3 cells and a significant increase in PGCC formation in OVCAR3 cells.

Taken together, two clinically-available inhibitors of autophagy did not prevent the formation of PGCCs in OVCAR3 or CAOV3 ovarian cancer cells. These autophagy inhibitors worked differently than CPt or DTx in that neither autophagy inhibitor stressed CAOV3 or OVCAR3 cells to induce PGCC formation, although in combination with chemotherapy there was a trend toward increased PGCC formation.

### The autophagy activator rapamycin does not exacerbate PGCC formation

**Fig. 5:**
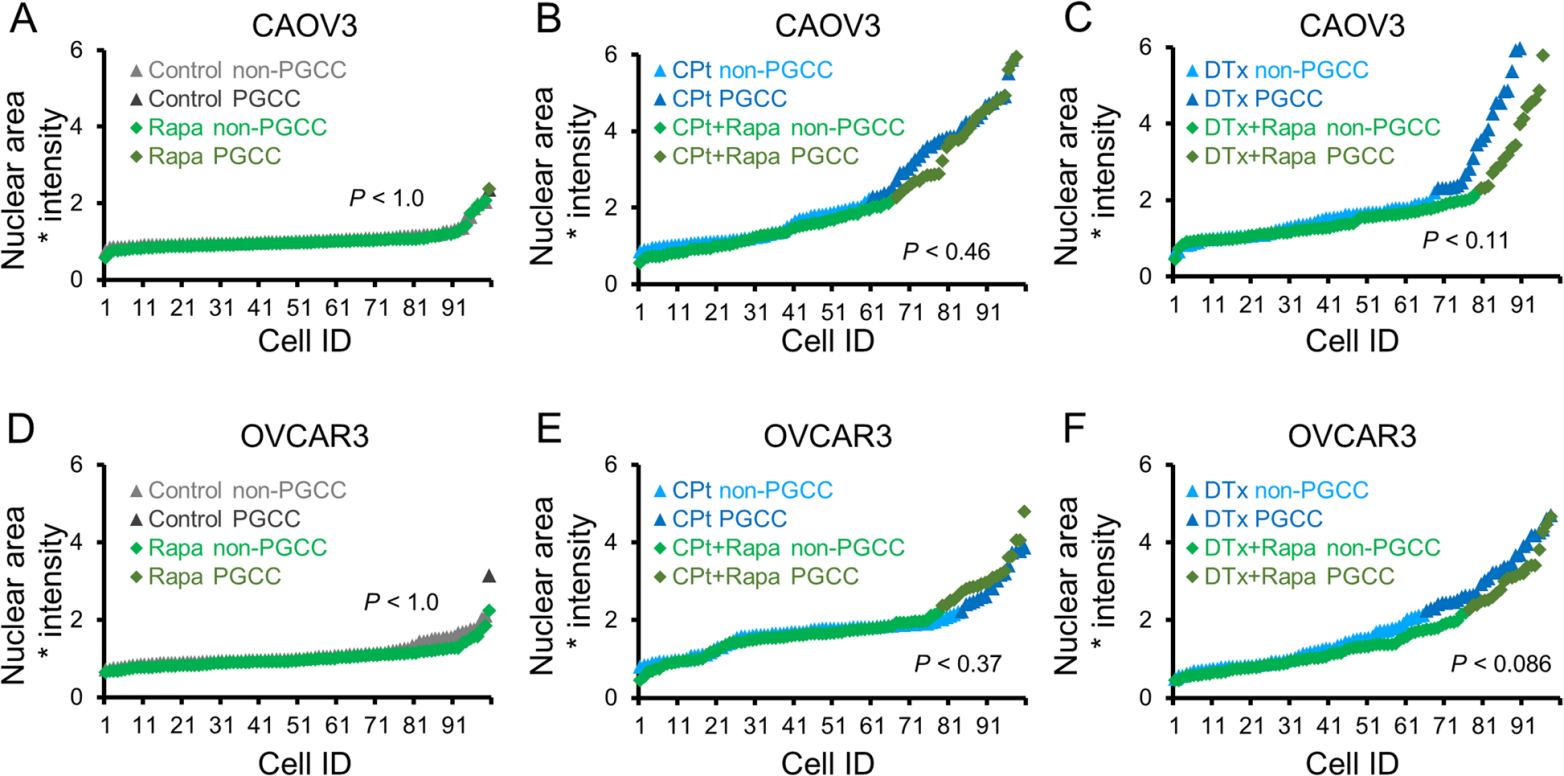
Rapamycin (Rapa) does not increase PGCC formation in CAOV3 or OVCAR3 cells.** A** CAOV3 cells were treated with 10 nM Rapa or vehicle control for 72 h and allowed to recover in fresh drug-free media for 72 h prior to PGCC quantitation by Hoescht 33342 staining. **B** CAOV3 cells were co-treated with CPt and Rapa or vehicle control for 72 h and allowed to recover in fresh drug-free media for 72 h prior to PGCC quantitation. **C** CAOV3 cells were co-treated with DTx and Rapa or vehicle control for 72 h and allowed to recover in fresh drug-free media for 72 h prior to PGCC quantitation. **D-F** Identical experiments as in (**A-C**) were performed using OVCAR3 cells. *P*-values indicated Fisher’s exact test result, and 100 cells quantified from two independent experiments are shown

Since autophagy is induced during PGCC formation, it is reasonable to hypothesize that further stimulation of autophagy might enable more cells to form PGCCs following chemotherapy induction. To test this, the well-characterized, clinically available autophagy inducer rapamycin (Rapa) was used. Rapa treatment was conducted in the context of PGCC formation by CAOV3 and OVCAR3 cells. Rapa alone had no significant effect on PGCC formation of CAOV3 cells (Fig. 5A). In CAOV3 cells co-treated with CPt and Rapa versus CPt alone, an insignificant decrease in PGCCs to 34% from 40% was observed (Fig. 5B). Similarly, co-treatment of CAOV3 cells with Rapa and DTx versus DTx alone, a decrease to 21% from 32% PGCCs respectively was observed, but this trend did not reach significance (*p* = 0.11; Fig. 5C). Rapa alone had no significant effect on PGCC formation of OVCAR3 cells (Fig. 5D). OVCAR3 cells displayed no significant difference in PGCC formation when co-treated with Rapa and CPt compared to CPt alone (Fig. 5E). In OVCAR3 cells co-treated with Rapa and DTx versus DTx alone, a decrease to 23% from 35% PGCCs was observed, but this difference was not statistically significant (Fisher’s exact test statistic 0.086; Fig. 5F). Thus, although Rapa tended to decrease chemotherapy-induced PGCC formation in both CAOV3 and OVCAR3 cells, these trends were not statistically significant. Taken together with the HCQ and NFV results, these findings are inconsistent with the hypothesis that autophagy induction is either necessary or sufficient for PGCC induction.

### Autophagy-targeting therapeutics affect expression of the autophagy markers GRP78 and LC3B

**Fig. 6:**
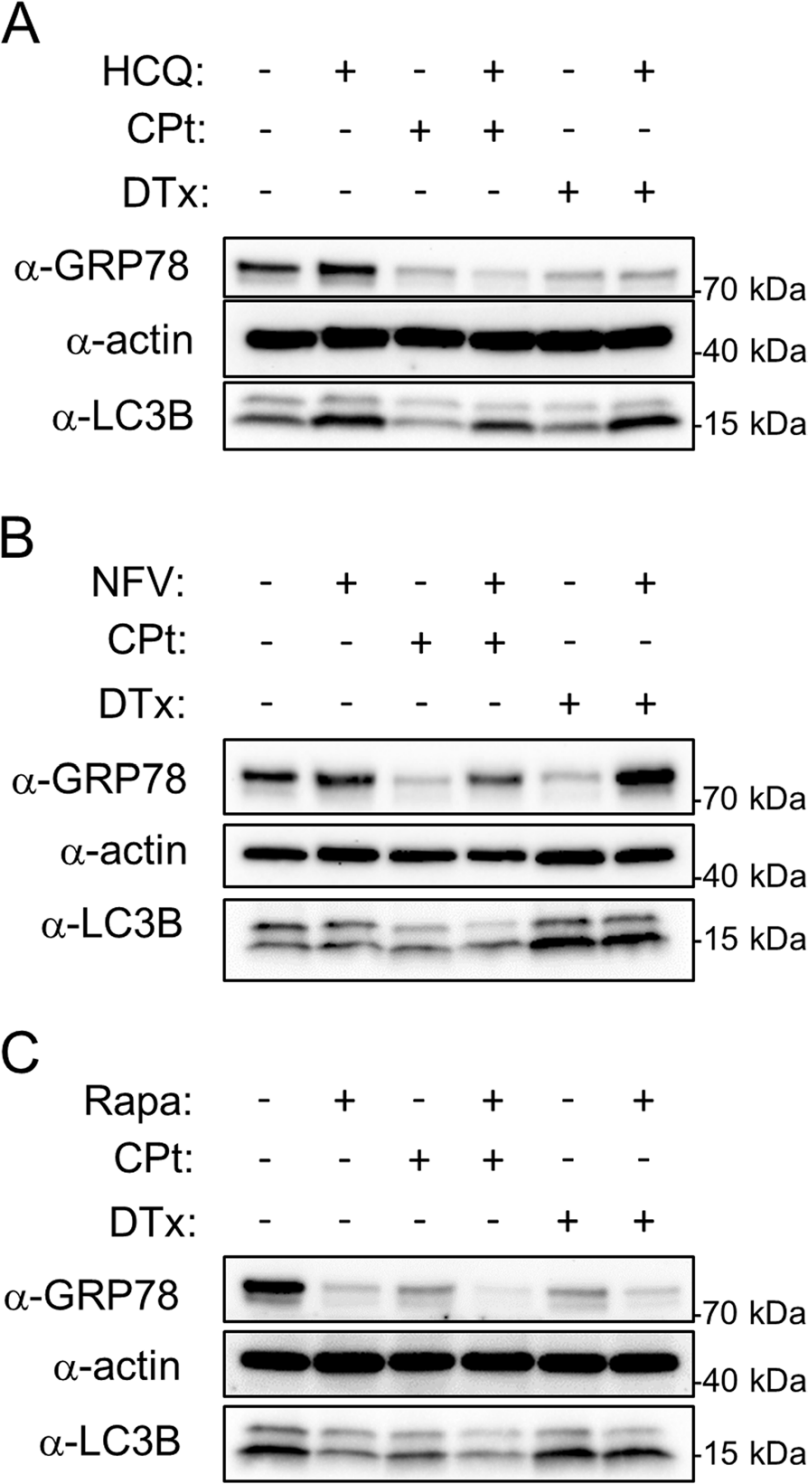
Autophagy-modulating therapeutics alter the expression of GRP78 and LC3B-II. CAOV3 cells were treated for 72 h with vehicle control or HCQ (**A**), NFV(**B**), or Rapa (**C**) and concurrently with and without CPt or DTx as indicated and western blotting for GRP78, actin, and LC3B was conducted as described in Methods. LC3B runs as a doublet; the upper band is free cytosolic LC3B-I and the lower band is lipidated LC3B-II present in autophagosomes. Blots are representative of two independent experiments. Full-length blots are presented in Supplementary Fig. 1

Western blotting was employed to confirm that the autophagy markers GRP78 and LC3B were affected by treatment with the autophagy-modulating therapeutics. CAOV3 cells were treated, as in Figs. 3, 4 and 5, with 33 µM HCQ, 10 µM NFV, or 10 nM Rapa both without and with cotreatment with CPt or DTx. After 72 h of treatment, the cells were harvested for western blotting of β-actin, GRP78, and LC3B as described in Methods. HCQ inhibits autophagosomal-lysosomal fusion resulting in the accumulation of autophagosomes. As expected, HCQ treatment alone or in concert with CPt or DTx consistently increased LC3B-II – the lower band consists of lipidated (conjugated to phosphatidylethanolamine) LC3B, referred to as LC3B-II, which is present in autophagosomes (Fig. 6A). HCQ treatment did not have a marked effect on GRP78 levels, although HCQ alone slightly increased GRP78 expression (Fig. 6A). NFV treatment did not have a marked effect on LC3B-II levels, but NFV, especially NFV cotreatment with CPt or DTx, led to an increase in GRP78 levels (Fig. 6B). Treatment with the autophagy activator Rapa, by contrast, led to a marked decrease in GRP78 levels whether used alone or in concert with CPt or DTx chemotherapies, and Rapa treatment resulted in decreased LC3B-II (Fig. 6C). Together these results demonstrate that the doses of the autophagy-modulating therapeutics employed here affected autophagy in the expected manner.

### Autophagy disrupting drugs prevent tumor cell colony formation from isolated PGCCs

Given that the autophagy drugs we tested here ablate HGSC tumors *in vivo* [18], we postulated that PGCC life cycle must be disrupted at a stage other than initiation. Therefore, we tested the effect of these same autophagy modulators on the next stage of PGCC life cycle: tumor cell colony formation. CPt and DTx were used to induce PGCC formation in CAOV3 and OVCAR3 cells as above. On day 7, PGCCs were filter-purified and re-plated. Isolated PGCCs were then treated with HCQ, NFV, or Rapa for 6 days (days 8–14), during PGCC daughter cell formation. On day 14, a total of 10 days after the end of chemotherapy exposure, colonies of PGCC progeny were stained with crystal violet and representative images were captured before re-suspending the crystal violet and quantifying absorbance at 600 nm (as described in Methods and Fig. 1C).

**Fig. 7:**
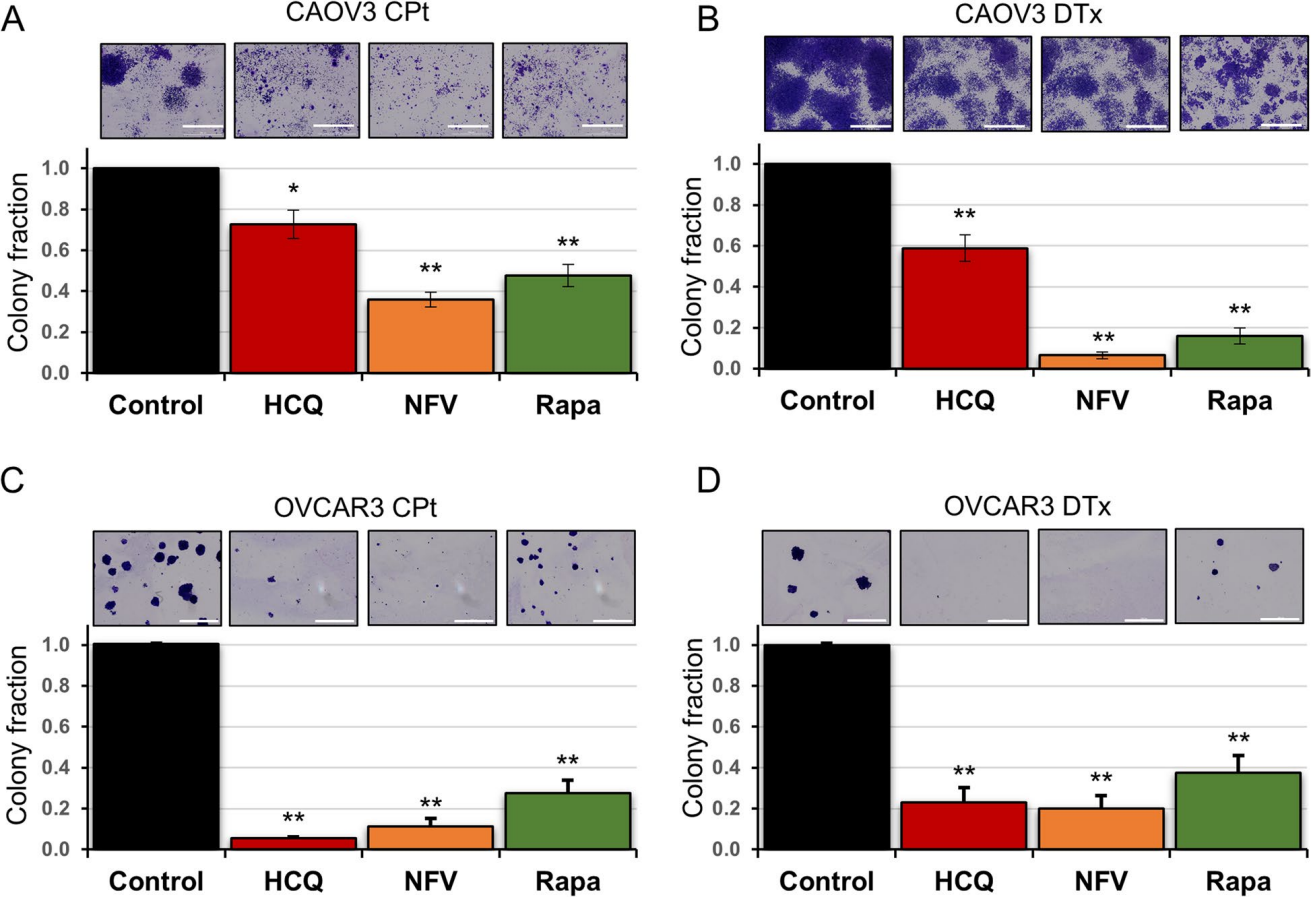
HCQ, NFV, and Rapa prevent colony outgrowth from chemotherapy-induced PGCCs in CAOV3 and OVCAR3 cells.** A** CAOV3 PGCCs were initially formed using the 72 h CPt treatments followed by fresh drug-free media for 72 h. These PGCCs were isolated using size-exclusion filters and replated. 33 µM HCQ, 10 µM NFV, or 10 nM Rapa was then added and replenished 72 h after first drug addition. Resultant colonies originating from isolated PGCCs were then stained and quantified using crystal violet. Representative stained colonies are shown. **B** A similar assay was used to query CAOV3 PGCCs formed by 5 nM DTx. **C, D.** Identical assays as in (**A**, **B**) were performed for OVCAR3 cells. Data are mean ± standard deviation of three independent experiments. Scale bar indicates 2 mm. * *P* < 0.05 and ** *P* < 0.01 by Student’s t-test comparing drug-treated to vehicle control

Treatment of CAOV3 cells with HCQ during PGCC progeny formation led to a significant reduction in progeny formation to 73% of control for PGCC progeny formed following exposure to CPt and 59% of control for PGCC progeny formed following DTx exposure (Fig. 7A and B). A decrease in CAOV3 PGCC progeny was also observed following treatment with NFV where only 36% CPt-induced PGCC progeny and 6% DTx-induced PGCC progeny formed (Fig. 7A and B). In OVCAR3 cells, treatment with the same dose of HCQ led to a larger reduction of PGCC progeny formation; HCQ treatment reduced progeny by 95% in the context of CPt and 77% in the context of DTx-induced PGCC daughter cell formation (Fig. 7C and D). Likewise, treatment with NFV decreased progeny formation by OVCAR3 PGCCs by 89% for CPt-induced PGCCs and 80% for DTx-induced PGCCs (Fig. 7C and D). Taken together, the prevention of PGCC-mediated colony formation by clinically available autophagy inhibitors was observed.

Similar to our PGCC formation rationale, we suspected the autophagy activator Rapa may have the opposite effect of HCQ and NFV. Rapa was next investigated in the context of PGCC-mediated colony formation. Surprisingly, Rapa decreased progeny generation by CAOV3 CPt-PGCCs by 50% and ~ 80% for DTx-PGCCs (Fig. 7A and B). In OVCAR3 cells, Rapa treatment decreased PGCC progeny generation from CPt-induced PGCCs to 28% of controls and DTx-induced PGCC progeny formation to 37% of controls (Fig. 7C and D).

## Discussion

Two decades of research have well-established that autophagy plays a role in chemoresistance. Remarkably, none of these previous studies have tested if autophagy inhibitors modulate the PGCC life cycle, despite the clear formation of these cells following chemotherapy and radiotherapy. Although it is known that autophagy is elevated in PGCCs [24, 25], to our knowledge this is the first study which directly examines the interaction of autophagy modulating drugs and PGCCs. We observed that while autophagy inhibitors do not prevent PGCC formation, autophagy inhibitors are able to reduce the amount of progeny which arise specifically from PGCCs. Given that PGCCs are a source of chemoresistant cancer cells and a source of random karyotype shuffling and therefore intra-patient genetic diversity, autophagy inhibitors may be promising to pursue in the clinic for HGSC patients exposed to carboplatin or docetaxel chemotherapy.

Exceptionally large cancer cells with large, abnormal nuclei have been described in the scientific literature since the 1850s (reviewed in [32]), and PGCCs are present at low levels in many cancer cell lines and in virtually all types of cancer including HGSC [8, 12, 13, 33, 34]. Cells with these abnormal morphologies were traditionally regarded as dying or irreversibly senescent, but there is growing recognition that some PGCCs are able to overcome senescent cell-cycle arrest and spawn near-diploid progeny, enabling cancer cells to survive senescence induced by therapy or other stresses encountered in the tumor microenvironment such as hypoxia or nutrient deprivation [35–37]. Further, the reversible polyploidization process facilitates genome instability, which “underlies the hallmarks of cancer” [38]. The PGCC life cycle enables the development of aneuploidy and the myriad copy number alterations accompanying that process [34]. This genome shuffling yields karyotype diversity that is a substrate upon which selection acts during tumor evolution, especially during the development of drug resistance [39].

Here, CAOV3 and OVCAR3 ovarian cancer cell lines were treated with chemotherapies to induce the PGCC life cycle: formation of PGCCs and subsequent neosis and colony formation by PGCCs. Autophagy-modulating therapeutics were added either during PGCC formation or during the time that PGCCs were producing progeny by neosis. We found that these autophagy-modulating therapeutics had minimal effects or actually increased the number of PGCCs that were formed in response to CPt and DTx. In marked contrast, treatment with autophagy-modulating therapeutics had a strong inhibitory effect on colony formation after PGCCs were already formed. Breast cancer PGCCs display elevated markers of autophagy LCB-II and p62/SQSTM1 but low autophagic flux, whereas progeny derived from these PGCCs have elevated rates of autophagy [25]. Elevated autophagy during PGCC progeny generation may be necessary to rid cells of irreversibly damaged DNA, organelles, and proteins. The lack of an effect of autophagy-modulating therapeutics on chemotherapy-induced PGCC formation and the inability of these therapeutics to effect basal PGCC levels suggests that autophagy is not critical for PGCC generation. In contrast, during neosis autophagy is elevated and treatment with the autophagy-modulating therapeutics HCQ or NFV significantly decreased PGCC progeny survival.

The similar effect of Rapa on reduced colony formation post-PGCC formation can be interpreted in a few ways, based on previous literature. The first, autophagy-independent explanation would be that Rapa inhibits cell growth processes via mTORC1 inhibition, resulting in fewer daughter cells. Previous observations make this interpretation somewhat unlikely; cell growth inhibition using this same dose of rapamycin in a panel of ovarian cancer cells exhibited 10–30% reduction in growth rates [18, 26], not the 52–84% inhibition of colony formation observed in the current study. However, autophagy-independent roles of mTORC1 may nonetheless be uniquely important after PGCCs have formed and start to re-seed tumors. The second interpretation is that Rapa creates a stress on autophagy, just as HCQ and NFV stress autophagy, so the similar effects would not be surprising. This is consistent with the observation that treatment with all three drugs results in HGSC accumulating aberrant vesicles and proteotoxic aggregates, as observed by electron microscopy [18]. This model further explains why Rapa actually worsens cytotoxicity of chloroquine and NFV, rather than ameliorating cell death caused by their administration. Future studies are warranted to better understand the molecular mechanism of these observations.

Aneuploidy was first hypothesized to cause cancer over a hundred years ago, but the discovery of oncogenes and tumor suppressor genes led to a “gene-centric” view of the etiology of cancer [40]. More recently, however, it has been suggested that a “genome-centric” view may be similarly appropriate [39]. Chromosomal instability is a high rate of chromosome mis-segregation that gives rise to aneuploidy. Aneuploidy is a hallmark of cancer, and ~ 90% of solid tumors have some degree of aneuploidy at the level of whole chromosomes [40, 41]. In addition, chromosome arm-level alterations and more focal copy number alterations are common in cancer and these chromosomal alterations and are included here in the term ‘aneuploidy.’ In most contexts, aneuploidy is associated with substantial fitness costs, but the pervasiveness of chromosomal instability and aneuploidy in cancers suggests that aneuploidies drive tumorigenesis, presumably by increasing genetic diversity. It is suggested that whole-genome duplications often precede the development of aneuploidy [42–46]. Whole genome-duplications are present in 37% of all cancers and 53–56% of HGSC [5, 6]. Both ovarian cancer cell lines used here, CAOV3 and OVCAR3, exhibit a hypotriploid karyotype, indicating that they likely evolved from a whole genome doubling event followed by chromosome loss [47]. HGSC is characterized by extensive aneuploidies, and the degree of aneuploidy correlates with malignancy and poor prognoses [48–50]. Due to extensive aneuploidy including focal copy number alterations, ~ two-thirds of genes are altered in dosage in a typical HGSC tumor [18]. The autophagy pathway is the most downregulated pathway by copy number alterations in HGSC with 95% of tumors having multiple heterozygous deletions in at least four autophagy genes. In addition, loss of autophagy genes in HGSC is shown to cause genomic instability [51]. Further, a cocktail of drugs including chloroquine, NFV, and Rapa which affect several nodes in the autophagy pathway simultaneously demonstrated remarkable efficacy in killing ovarian cancer cells in preclinical studies [26]. By virtue of having reduced capacity for functional autophagy, it appears that ovarian cancer cells have a unique vulnerability to drugs targeting this pathway.

It is generally accepted that tumor cell populations evolve, and that intra-tumor genetic heterogeneity is one source of variation upon which selection acts. In addition to genetic diversity at the level of gene mutations, genetic diversity arises from genomic diversity caused by different aneuploidies, and tumor heterogeneity also manifests at the epigenetic and phenotypic levels. Initially in tumorigenesis, gradual clonal evolution of a population of cells with various oncogene or tumor suppressor gene mutations results in sustained proliferation and decreased responsiveness to DNA damage as well as other cancer hallmarks. Next, excessive endogenous stress in the tumor microenvironment or stress induced by chemotherapeutics may lead to chromosomal instability, causing extensive aneuploidies which change the expression levels of thousands of genes in one generation. These profound chromosomal alterations may enable rapid punctuated evolution of cancer genomes and be instrumental in tumor progression, including the development of chemo resistant relapsed disease. The polyploidization process may underlie the ability of cancers to evolve resistance to virtually all current therapies. Following genome doubling, more potential for beneficial mutations is created because extra intact copies of genes are available if an allele is mutated deleteriously. Further, there is more genomic material available to participate in DNA repair processes and having additional copies of genes can result in increased expression of proteins involved in stress responses. These attributes facilitate the survival and evolution of cancer cell populations.

## Conclusions

Autophagy may present an Achilles heel to PGCCs for several reasons. Autophagy is induced during senescence, and autophagy may provide a vital function during senescence such as by recycling damaged cellular constituents. Proper regulation of autophagy may be necessary for PGCCs to produce viable progeny. The proper regulation of autophagy during the PGCC life cycle may be essential for cancer resurgence, and the data presented here using clinically available inhibitors of autophagy suggest that this is indeed the case. In sum, autophagy appears to present a viable therapeutic target to prevent the deadly PGCC tumor repopulation process.

## Supplementary Information

**Additional file 1: Figure S1.** Uncropped western blots to accompany Fig. 6. Red lines denote membrane cuts prior to antibody administration. Dotted line groups denote different exposures of the same blot.

## Data Availability

The datasets used and/or analysed during the current study are available from the corresponding author on reasonable request.

## Abbreviations

CNA: Copy number alteration
CPt: Carboplatin
DTx: Docetaxel
HCQ: Hydroxychloroquine
HGSC: High-grade serous ovarian carcinoma
NFV: Nelfinavir mesylate
PGCC: Polyploid giant cancer cell
Rapa: Rapamycin
