# Supplementary figures and images for: Autophagy modulating therapeutics inhibit ovarian cancer colony generation by polyploid giant cancer cells (PGCCs)
Source: BMC Cancer. 2022 Apr 14;22:410. doi: 10.1186/s12885-022-09503-6 (PMC9012005; doi:10.1186/s12885-022-09503-6)

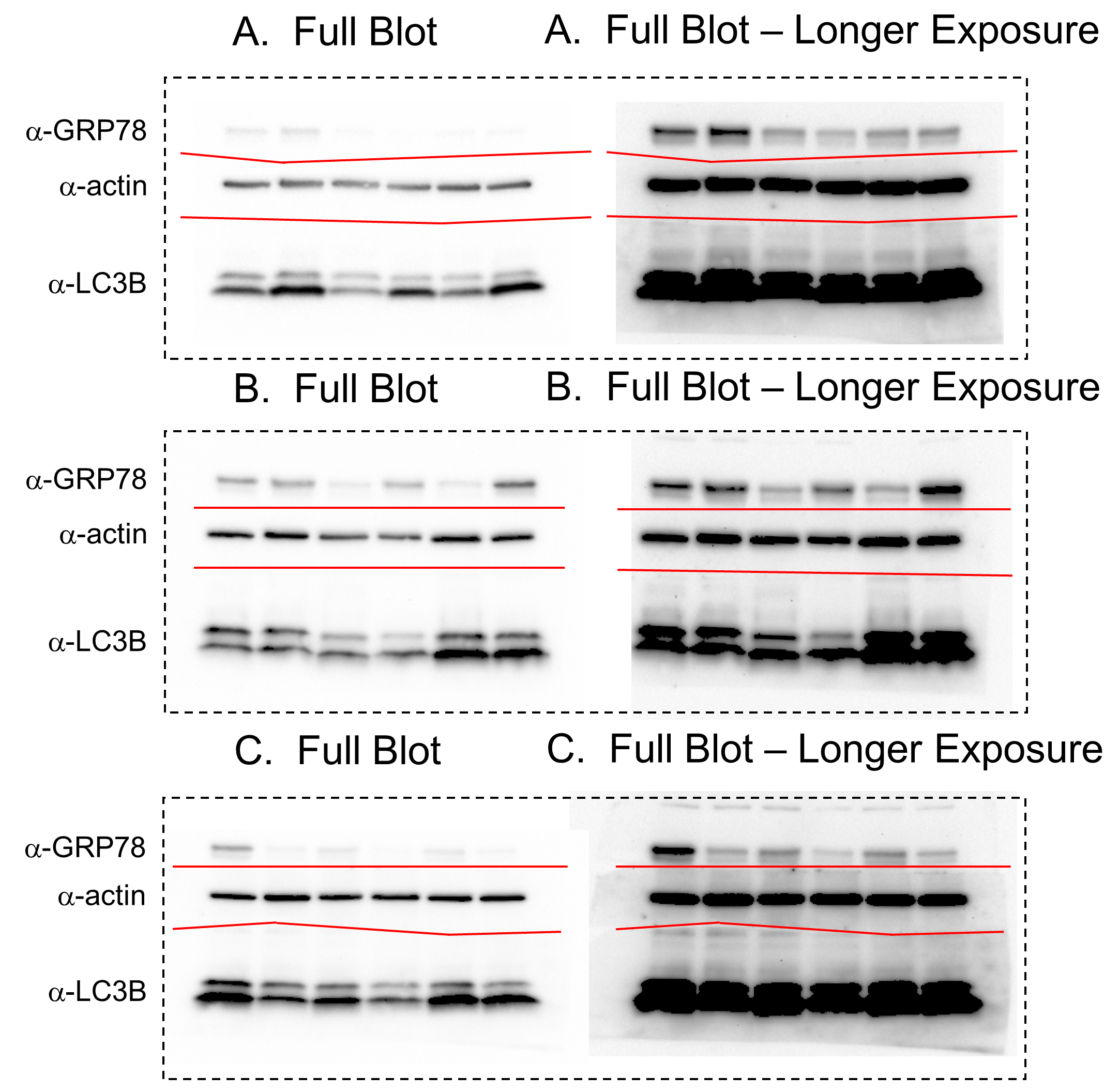

Supplement: Supplementary file 1 — Additional file 1: Figure S1. Uncropped western blots to accompany Fig. 6. Red lines denote membrane cuts prior to antibody administration. Dotted line groups denote different exposures of the same blot. [file 12885_2022_9503_MOESM1_ESM.tif]
